# Immune dysregulation in Periodic Fever, Aphthous atomatitis, Pharyngitis, Adenitis (PFAPA) syndrome

**DOI:** 10.1186/1546-0096-13-S1-P196

**Published:** 2015-09-28

**Authors:** L Broderick, D Carvalho, A Magit, W Jiang, S Leuin, M Bothwell, D Kearns, S Pransky, H Hoffman

**Affiliations:** 1 University of California, San Diego, La Jolla, San Diego, CA, USA; 2 Rady Children's Hospital-San Diego, San Diego, CA, USA

## Introduction

Periodic fever, aphthous stomatitis, pharyngitis and adenitis (PFAPA) syndrome is an autoinflammatory disorder of childhood and little is known about the underlying etiology, pathogenesis or the reason behind the success of tonsillectomy. Numerous immune cell types are present in the tonsillar microenvironment, each of which may contribute to the pattern of symptoms observed in PFAPA patients.

## Objectives

To identify differences in cellular populations in patients with PFAPA syndrome compared to controls with recurrent pharyngitis and obstructive sleep apnea, and analyze if these cells have a proinflammatory phenotype.

## Methods

Patient data and detailed family histories were collected for over 200 children with recurrent fevers including 94 patients with PFAPA to create a prospective cohort of children treated at a tertiary care center in San Diego, CA, USA. Patient data was collected under an IRB-approved protocol using patient charts and a standardized questionnaire, demographic data, including age, gender, and ethnicity, clinical profiles (presence of symptoms, fever profile, treatments) and detailed family histories over a 7-year period.

For patients electing to undergo tonsillectomy (n = 63), whole tonsillar tissue was obtained post-operatively under an IRB-approved protocol. Control tonsils (n = 22) were obtained from children of similar age with either recurrent streptococcal pharyngitis or obstructive sleep apnea. Single cell suspensions were derived and fluorescently stained for evaluation by eight-color flow cytometry. In some cases, cells were cultured with LPS or CpG *in vitro*, prior to flow cytometric analysis.

## Results

Flow cytometry of isolated cellular constituents reveals that tonsils from patients with PFAPA have a significant memory B cell population, defined as CD27+, CD19+, CD3 negative cells (p<0.01), with similar T cell, NK cell and monocyte/macrophage populations. PFAPA patient tonsillar memory B cells express higher levels of the survival markers BAFF-R and TACI and significantly more intracellular IL-1b, compared to memory B cells from controls (p<0.05). Activation of tonsillar cells in culture with lipopolysaccharide (LPS) significantly increases the percent of IL-1b positive cells in control cultures (p<0.01), but has no effect on cells derived from PFAPA patients. Similarly, stimulation with CpG failed to further upregulate BAFF-R and TACI expression on PFAPA tonsillar memory B cells to the extent observed in stimulated control cultures.

## Conclusions

Taken together, these findings suggest that PFAPA patient tonsillar cells are constitutively activated or primed for hyperresponsiveness even during afebrile periods.

